# Instant green synthesis of silver-based herbo-metallic colloidal nanosuspension in *Terminalia bellirica* fruit aqueous extract for catalytic and antibacterial applications

**DOI:** 10.1007/s13205-016-0589-1

**Published:** 2017-04-13

**Authors:** Sandeep Patil, Gunjan Chaudhari, Jayasinh Paradeshi, Raghunath Mahajan, Bhushan L. Chaudhari

**Affiliations:** 1 Department of Microbiology, School of Life Sciences, North Maharashtra University, Umavi Nagar, Post Box 80, Jalgaon, 425 001 India; 2 Department of Biochemistry, Moolji Jaitha College, Jalgaon, 425002 India; 3 Post Graduate College of Science Technology and Research, Moolji Jaitha College Campus, Jalgaon, 425002 India

**Keywords:** Silver nanoparticles, Instant green synthesis, *Terminalia bellirica*, Catalysis, Antibacterial, Antibiofilm

## Abstract

In the present study, microwave-assisted, optimized, instant, *Terminalia bellirica* fruit extract-mediated green synthesis of colloidal silver nanoparticles (AgNPs) has been reported. The synthesized AgNPs were characterized by UV–Vis spectroscopy, FTIR, Zetasizer, FESEM, EDX and XRD. The characteristic surface plasmon peak of reaction mixture at 406 nm confirmed the synthesis of AgNPs. The FTIR studies confirmed phytoconstituents were responsible for the synthesis and stability of AgNPs. The FESEM, EDX and XRD analysis revealed the presence of spherical silver nanoparticles of mean diameter ≤20.6 nm with face-centered cubic crystalline structure. These AgNPs showed notable catalytic activity in reduction of 4-nitrophenol to 4-aminophenol in the presence of NaBH_4_. The synthesized AgNPs showed potential antibacterial and antibiofilm activity against bacterial pathogens like *Bacillus subtilis*, *Escherichia coli*, *Pseudomonas aeruginosa* and *Staphylococcus aureus*. Thus, these synthesized AgNPs can open avenues for the development of AgNP-based efficient nanocatalyst and potent nanomedicine in future.

**Electronic supplementary material:**

The online version of this article (doi:10.1007/s13205-016-0589-1) contains supplementary material, which is available to authorized users.

## Introduction

Silver nanoparticles have found remarkable applications in the field of drug delivery, food industries, agriculture, textile industries, water treatment, redox catalysis, green housing construction and medicine (Jagtap and Bapat 2013; Kuunal et al. 2016). Several approaches exist for the synthesis of silver nanoparticles (AgNPs) including; thermal decomposition, sonochemical, electrochemical and photochemical reactions, chemical reduction and biological route (Ahmad et al. 2010). Physical and chemical methods could effectively produce pure and distinct nanoparticles; however, these methods are quite costly and possibly harmful to the environment due to use of harsh chemicals (Kumar and Yadav 2009). This necessitates cost-effective, commercially feasible, non-toxic and environment friendly process for the synthesis of AgNPs. Biological materials such as microbes, enzymes, plant materials, etc., offer ecofriendly approach for the synthesis of nanoparticles (Velmurugan et al. 2011). Synthesis of nanoparticles using microorganisms has limitations due to its slow rate of synthesis (Shahverdi et al. 2007); hence, plant-based materials are receiving more attention due to its simplicity, ready scalability, ecofriendliness, cost-effectiveness and relatively high reproducibility (Iravani 2011). The key active agents in such nanoparticles synthesis were speculated to be polyphenols, flavonoids, reducing sugars, sterols, essential oils, starch, cellulose, pectins, gums, resins, lectins, etc. These biomaterials act as reducing agents as well as capping agents in the synthesis of silver nanoparticles (Gangula et al. 2011).

In recent days there has been a growing interest in developing nanomaterial-based antimicrobial agents to combat the emerging resistance to antimicrobial agents by pathogenic bacteria (Seil and Webster 2012). Ability of bacterial pathogens to form biofilms offers 1000 times more resistance against antimicrobial agents (Mah and O’Toole 2001). Hence there is a necessity to develop antimicrobial agents which have broad-spectrum activity and potential to combat against antibiotics resistant biofilms.

The present work deals with instant green synthesis of biocapped AgNPs by using *Terminalia bellirica* (Roxb.) fruit aqueous extract. This plant is wild and grows throughout the Indian subcontinent, Nepal, Srilanka, Malaysia and South East Asia (Ramesh et al. 2005). In traditional Indian Ayurvedic medicine, *T. bellirica* fruit is used in the popular Indian herbal rasayana treatment triphala. *T. bellirica* is used to protect the liver, reduce high cholesterol, and treat digestive as well as respiratory disorders (Latha and Daisy 2011). It has a well-established antioxidant potential and presence of polyphenolic compounds such as ellagic acid, gallic acid, tannins, ethyl gallate, galloyl glucose, chebulagic acid, 7-hydroxy 3′4′ (methylene dioxy) flavones, etc., as well as reducing sugars such as glucose and rhamnose (Nampoothiri et al. 2011). Hence, this plant was chosen for the synthesis of AgNPs. To the best of our knowledge, the use of *T. bellirica* fruit aqueous extract has not been reported before for the synthesis of AgNPs.

In these studies, the microwave-assisted rapid synthesis of colloidal AgNPs using *TB* extract has been reported. The process variables such as the relative concentrations of the extract and metal salt(s) in reaction mixture, pH, and time of reaction which controls the key properties of nanoparticles have been optimized. Furthermore, applicability of these AgNPs as a nanocatalyst in the reduction of 4-nitrophenol was explored. Besides biomedical application of these AgNPs such as antibacterial and antibiofilm agents against human pathogenic bacteria were also assessed.

## Materials and methods

### Chemicals and collection of plant material

Chemicals such as silver nitrate, sodium borohydride, 4-nitrophenol used in this research work used were of high grade and purchased from HiMedia, Mumbai. The dried fruits of *T. bellirica* were collected from local market and are available throughout India. The plant material was authenticated by an expert botanist.

### Preparation of aqueous extract of *Terminalia bellirica* fruit

The dried fruits of *T. bellirica* were cleaned with distilled water, shade dried, and ground to a fine powder and then sieved through 60 mesh size sieve. The aqueous extract was prepared by boiling under pressure in an autoclave which involved the addition of 20 g of powdered fruits with 200 mL of distilled water, autoclaved for 12 min at 121 °C under pressure 15 psi. Further, the extract was centrifuged at 10,000 rpm for 15 min and then filtered through a membrane having 0.2 µm pore size. The filtrate was stored in the refrigerator at 4 °C until its use. The dry weight of *TB* extract per mL of filtrate was determined. Total phenolic content, total flavonoid content, total reducing sugars and total reducing capacity of *TB* extract were determined using colorimetric assays (Wojdyło et al. 2014).

### Synthesis of AgNPs

The synthesis of AgNPs was carried out in two different sets each having 100 mL of 3 mM AgNO_3_ solution with 1.5 mL of extract. In the first set AgNP synthesis was monitored under normal conditions at room temperature and in another set the reaction mixture was irradiated in a domestic microwave oven (GMS 17M 07 WHGX Godrej, India), at working frequency 2450 MHz and power output 900 W, for 5 min. For both the sets, separate controls were run without addition of extract to 100 mL of 3.0 mM AgNO_3_ solution. Formation of silver nanoparticles was visually observed by color change of the reaction mixtures as well as scanning UV–Vis spectra of it from 250 to 750 nm range using UV–Vis spectrophotometer (UV-1800, Shimadzu, Japan), in both the sets. The samples were diluted twofold by deionized distilled water for UV–Vis spectral analysis. Process parameters involved in the synthesis of AgNPs such as pH of the reaction mixture, ratio of concentration of *TB* extract with AgNO_3_ solution and time of microwave irradiation were optimized by one factor at a time method (Online Resource).

### Spectroscopic and microscopic characterization of synthesized nanoparticles

Synthesis of AgNPs by reducing Ag^+^ ion solution with *TB* extract may be easily monitored by UV–Vis spectroscopy. Using a UV–Vis spectrophotometer (UV-1800, Shimadzu, Japan) absorption spectra were measured in the 250–750 nm range against deionized distilled water as blank. In order to determine the involvement of bioactive functional groups in reduction, capping and stabilization of Ag^+^ ions, Fourier transform infrared (FTIR) spectra of *TB* extract and AgNPs were recorded by KBr pellet method on FTIR spectrometer (Spectrum Two, FTIR-88522, Perkin Elmer, USA). Average particle size and stability of green synthesized AgNPs were analyzed using the Malvern Zetasizer (NanoZS-90, UK) instrument. The surface morphology and the presence of elemental silver in green synthesized AgNPs were analyzed by field emission scanning electron microscopy (FESEM) and energy-dispersive X-ray spectroscopy (EDX) using instrument FESEM (S4800 Type II, Hitachi, Japan) equipped with EDX (X Flash detector-5030, Bruker, Germany). To determine crystallinity X-ray diffraction (XRD) data were acquired by an X-ray diffractometer (Bruker; D8 Advance, Germany).

### Catalytic activity of AgNPs

The catalytic reduction reaction of 4-nitrophenol was carried out in aqueous solution. Initially, 5.0 mM 4-nitrophenol (5 mL) and 0.2 M NaBH_4_ (6.25 mL) were mixed in a 100-mL conical flask; the volume was adjusted to 50 mL with deionized distilled water to get overall concentrations of 4-nitrophenol and NaBH_4_ 0.5 and 25 mM, respectively. Immediately after change in color from light yellow to yellow green, the UV–Vis absorption spectra of the solution were recorded with a time interval of 1 min with a scanning range of 200–750 nm at 25 °C on UV–Vis spectrophotometer (UV-1800, Shimadzu, Japan). Similarly same sets of reaction were carried out separately with the addition of 0.25 mL of *TB* extract (1.5% v/v) and AgNPs before adjusting reaction volume to 50 mL.

### Assessment of biomedical applications of AgNPs

Antibacterial activity of phytosynthesized AgNPs was evaluated by agar well-diffusion method (Gupta et al. 2014) against the pathogenic bacteria *Pseudomonas aeruginosa* (ATCC 9027), *Escherichia coli* (ATCC 8739), *Staphylococcus aureus* (ATCC 6538) and *Bacillus subtilis* (ATCC 6633) which were available in our laboratory. In brief, test sample of 50 µL (pH 7.0) was loaded in each well and incubated at room temperature against the said pathogens spread on nutrient agar. Antibacterial activity was expressed in terms of the inhibition zone (in mm). Deionized distilled water was used as a negative control. The antibiotic streptomycin (20 µg mL^−1^), AgNO_3_ solution (1.25 mM) and *TB* extract (150 µg mL^−1^) were used as positive controls. Minimum inhibitory concentration (MIC) was determined by the standard micro-dilution method recommended by the Clinical Laboratory Standardization Institute (CLSI) guideline (CLSI 2008).

The antibiofilm activity of AgNPs was evaluated by using crystal violet microtiter plate assay (Gupta et al. 2014). The lowest concentration that produced maximum biofilm inhibition was considered to be the biofilm inhibitory concentration (BIC). The potential of AgNPs to disrupt the established biofilms was also evaluated by treating pre-formed biofilms with the AgNPs under nutrient-limited and nutrient-rich conditions as described by Bakkiyaraj et al. (2013). These experiments were performed three times, with replicates of six, and average values were calculated.

### Statistical analysis

All experiments related to phytochemical analysis were performed in replicates of six while others were performed in triplicates. Results were expressed as the mean ± standard deviation (SD). Origin Pro 8 statistical program was used for graph design and data analysis.

## Results and discussion

### Total phenolic, flavonoid and reducing sugars content of *TB* extract and its reducing power

Recently, interest of nanotechnology is focused towards the green synthesis of nanoparticles (Park et al. 2011). Therefore, discovering natural reducing agents; especially those of plant origin become crucial, including mainly polyphenols, flavonoids and reducing sugars (Iravani 2011). Presence of polyphenols in *T. bellirica* (Nampoothiri et al. 2011) provoked to explore its potential in green synthesis of AgNPs. The total phenolic, flavonoid and reducing sugars content of *TB* extract were measured to be 20.54 ± 1.02 mg mL^−1^ (gallic acid equivalent), 3.78 ± 0.06 mg mL^−1^ (rutin equivalent) and 10.10 ± 0.15 mg mL^−1^ (maltose equivalent), respectively. The reducing power of *TB* extract, which could serve as a potent bioreductant in synthesis of metallic silver nanoparticles, was comparable with that of chemical reducing agents like ascorbic acid (Online Resource Fig. S1) suggesting that the *TB* extract possessed a stronger electron donating capacity. Thus, owing to strong reducing capacity of *TB* extract, it was exploited as reducing agent in biomimetic synthesis of AgNPs.

### Synthesis of AgNPs using *TB* extract

**Fig. 1:**
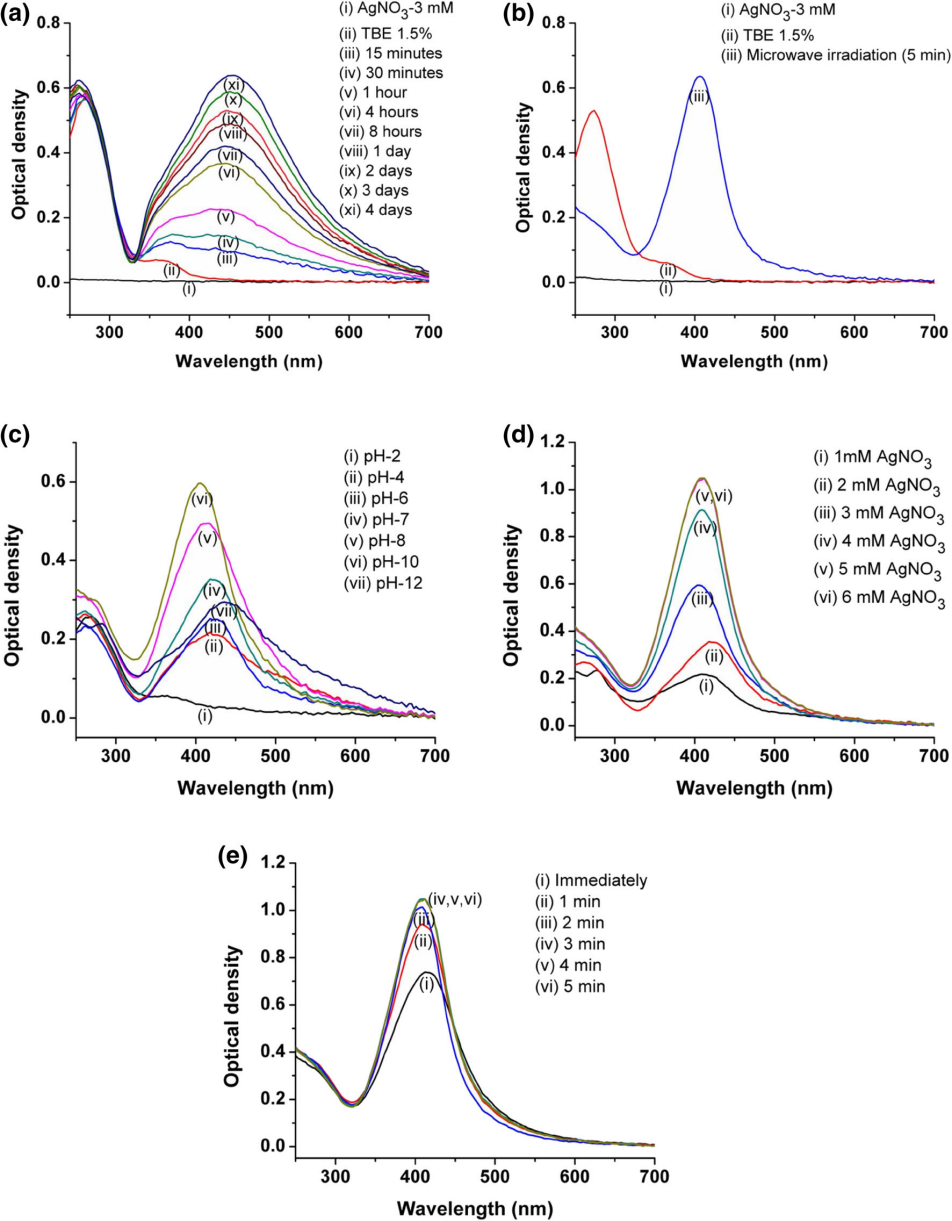
UV–Vis spectra of AgNP synthesis using *TB* extract: **a** under normal conditions; **b** under microwave irradiation; **c** at different pHs of reaction mixture; **d** at different concentrations of AgNO_3_ and **e** at different times of microwave irradiation

Synthesis of silver nanoparticles was easily determined and monitored by UV–Vis spectroscopic analysis due to their surface plasmon resonance phenomenon (SPR) (Kora et al. 2012). SPR is the interaction of electromagnetic radiation and the electrons in the conduction band around the nanoparticles (Ringe et al. 2010) giving well-defined absorption band in the visible region which is a manifestation of optical response of materials at different scales (Noguez 2007). The AgNPs show strong absorption peak in the range of 400–440 nm in a visible region (Shivaji et al. 2011). In the current work, after incubation of 100 mL of reaction mixture (pH 7) containing 3 mM AgNO_3_ and 1.5 mL of *TB* extract AgNPs were formed (Fig. 1a). However, it took longer time, about 4 days, for complete reduction of Ag^+^ to AgNPs. The AgNP synthesis was evident from the development of dark brown color (Online Resource Fig. S2) with its *λ*_max_ in the range of 400–450 nm. A typical brown colored silver solution was obtained due to excitation of the SPR in the metal nanoparticles. These results are in good agreement with the findings of Edison and Sethuraman (2012) in the plant-mediated synthesis of silver nanoparticles. Microwave irradiation is an advantageous approach as a part of green chemistry in plant extract-mediated instant synthesis of nanoparticles (Yallappa et al. 2013). In the present study, the irradiation in microwave oven has minimized the time of AgNP synthesis initially to 5 min which resulted in complete reduction of Ag^+^ and rapid synthesis of AgNPs (Fig. 1b). The AgNP synthesis under normal and microwave irradiation is a function of time (Online Resource Fig. S3); therefore, with increase in time of incubation or irradiation up to complete reduction of Ag^+^ to AgNPs there was an increase in SPR. *TB* extract-mediated synthesis of AgNPs was an instant green process involving direct interaction of silver ions with plant extract in presence of microwave radiations without any byproduct. Similarly, this process did not require any chemical stabilization material because plant secondary metabolites mainly polyphenols create robust coating over nanoparticles making them stable against aggregation (Kumar and Yadav 2009). Moreover, the rate of synthesis of silver nanoparticles was very high (within 5 min), which supports the use of plants over microorganisms in biological synthesis methods (Iravani 2011). In the current research work, the optimum parameters for the green synthesis were found to be pH 10 (Fig. 1c), AgNO_3_ concentration 5 mM (Fig. 1d) with *TB* extract concentration 1.5% (v/v) of reaction mixture, and microwave irradiation for 3 min (Fig. 1e). The optimized process variables supported the maximum synthesis of AgNPs with smaller particle size and having stability in very short time. These results are in agreement with the earlier findings of Krishnaraj et al. (2012).

### Spectroscopic and microscopic characterization of green synthesized AgNPs

**Fig. 2:**
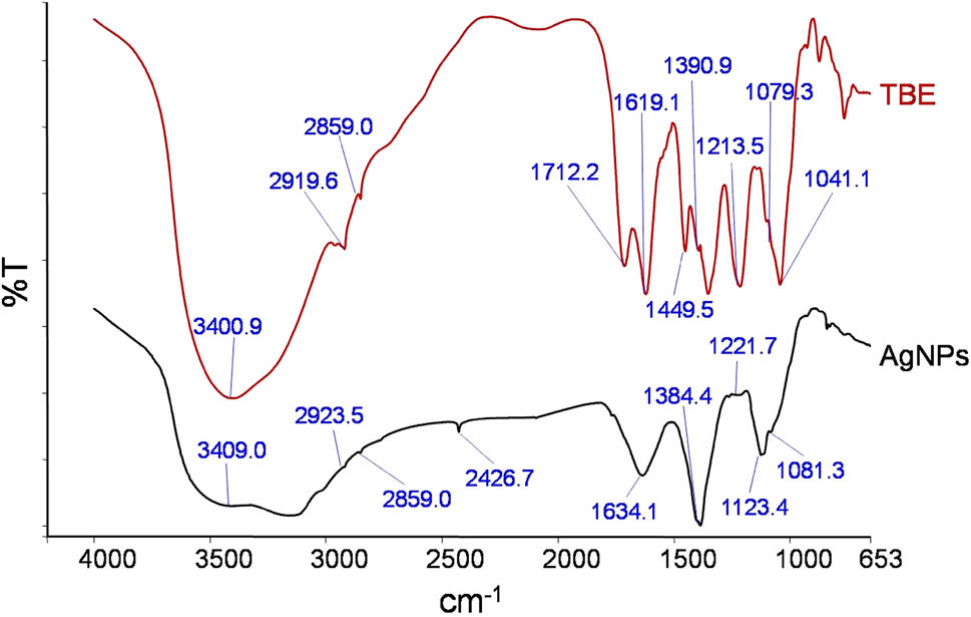
FTIR spectra of *TB* extract and biosynthesized AgNPs showing involvement of polyphenols in AgNP synthesis

In the current research work, the AgNPs were rapidly formed at pH 10 after the addition of *TB* extract, obvious from the appearance of dark brown color from pale yellow color with strong absorbance peak at *λ*_max_ 412 nm (Fig. 1b). The IR spectra identify the possible functional groups responsible for the reduction of ions and also the capping agents responsible for the stability of the biogenic nanoparticles (Thirunavukkarasu et al. 2012). In the present work, FTIR spectra of plant extract (*TB* extract) and biosynthesized AgNPs were analyzed (Fig. 2). IR spectrum of *TB* extract showed a characteristic peak at 3400 cm^−1^ which represents –OH stretch of phenolic compounds. The signals at 2919 and 2855 cm^−1^ were aroused possibly by asymmetrical and symmetrical stretching vibrations of C–H groups such as CH_2_ and CH_3_. The major peaks at 1712 and 1031 cm^−1^ corresponded to C=O stretch and C–O stretch of carboxylic acids, respectively. Peak at 1619 cm^−1^ was due to N–H bend of primary amines. The sharp peaks at 1431 and 1213 cm^−1^ indicated C–C stretch (in-ring) of aromatics and C–O stretch of esters. The band at 1041 cm^−1^ was related to C–N stretch of aliphatic amines. Absorption bands at 3400, 1712, 1634, 1213 and 1081 cm^−1^ appeared in FTIR spectrum of *TB* extract indicated the presence of polyphenolics such as gallic acid, ellagic acid, tannins, and ethyl gallate compounds (Vijayalakshmi and Ravindhran (2012). Similarly the peaks at 3400, 2919, 2855, 1712, 1619, 1390 and 1041 cm^−1^ are the characteristic peaks of lignocellulosic materials comprising reducing sugars as their building blocks (Sanchez et al. 2012) in *TB* extract. The absorption peaks that appear in the IR spectrum of *TB* extract could also be seen in the IR spectrum of green synthesized phytocapped AgNPs with minor variation in the positions of the absorption bands. This suggests the involvement of phytoconstituents in the synthesis of AgNPs and preventing them from aggregation.

### Particle size analysis and stability study

**Fig. 3:**
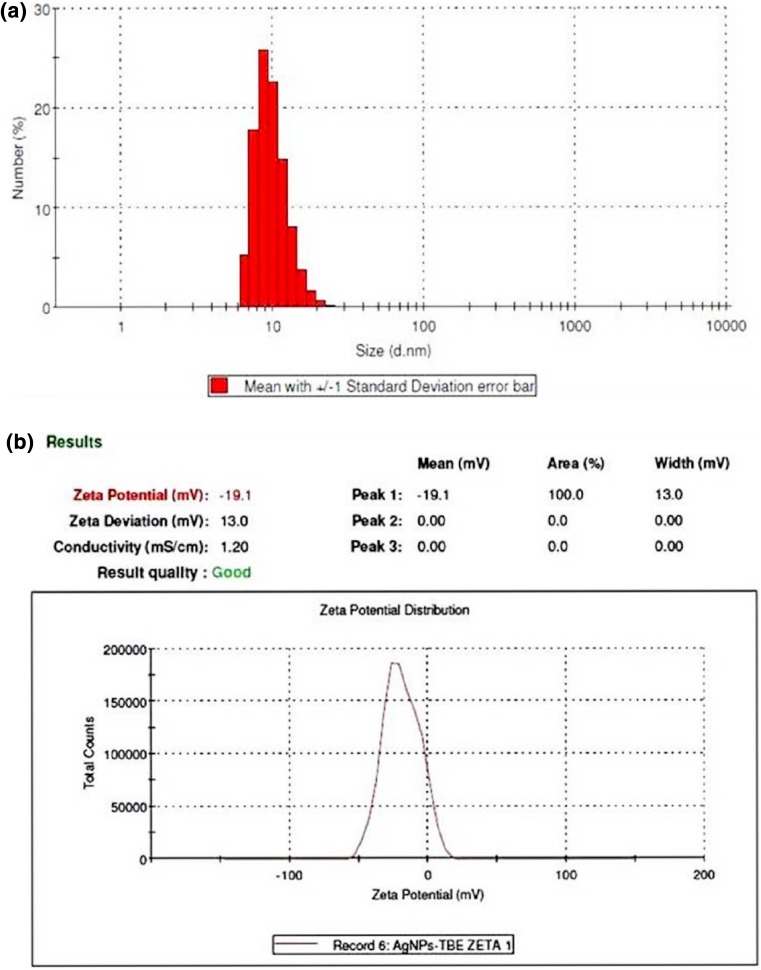
**a** Particle size distribution histogram and **b** Zeta potential measurement graph of biosynthesized AgNPs

In order to reveal the size of green synthesized AgNPs particle size analysis was performed in aqueous solution of AgNPs with Zeta analyzer. Particle size distribution histogram of AgNPs is shown in Fig. 3a. From these results, it was clear that the size of particles ranged from 6.50 to 24.36 nm with average size of 20.74 nm. These nanoparticles were having corresponding average zeta potential value −19.1 mV with good quality, indicating the stability of AgNPs (Fig. 3b). This significant negative potential value might be attributed to involvement of polyphenolic phytoconstituents for capping of nanoparticles (Vivek et al. 2012). Many plant extracts have been reported to have potential for controlled synthesis of AgNPs of varying morphologies and size distribution while Rauwel et al. (2015) reported that different plant extracts mediated synthesis of AgNPs having size from 2 to 100 nm and in the present work also the size of AgNPs was within similar range.

### SEM analysis of AgNPs

**Fig. 4:**
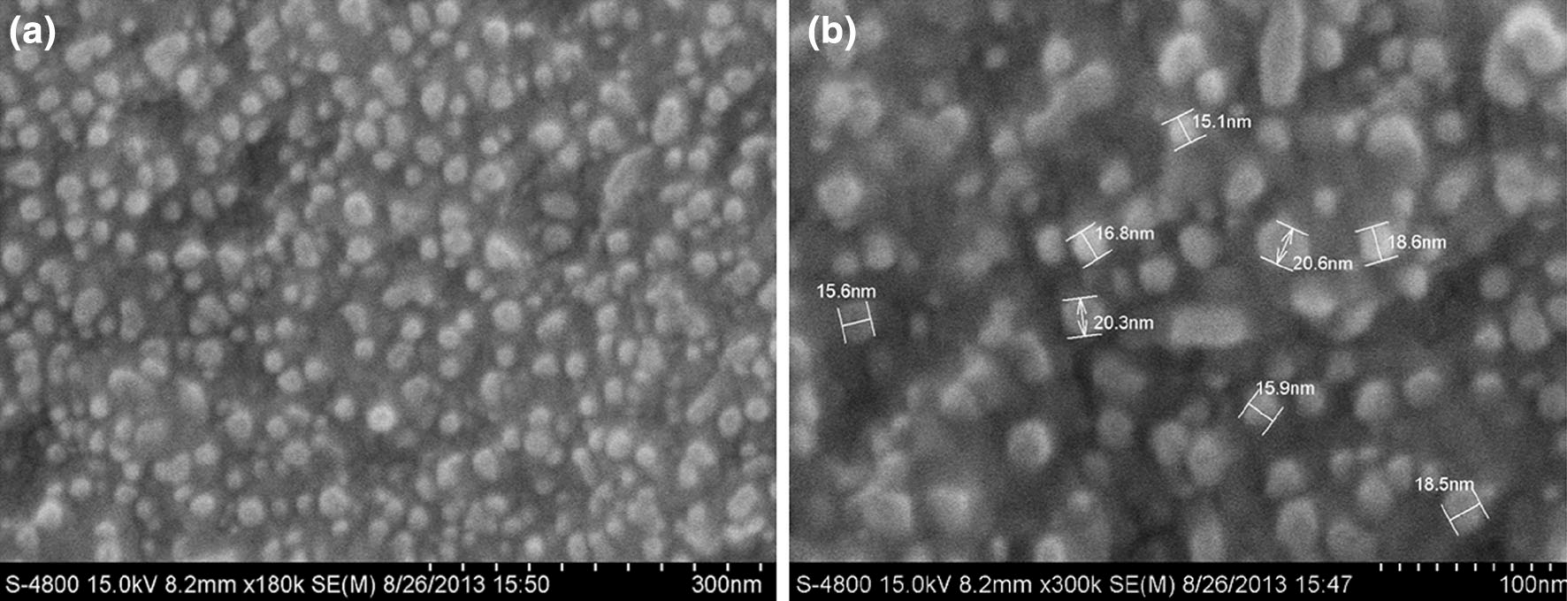
FESEM micrographs of the AgNPs formed after bio-reduction of AgNO_3_ by *TB* extract at magnifications **a** ×180k, **b** ×300k

The surface morphology of green synthesized AgNPs was investigated by FESEM analysis. These micrographs revealed more or less spherical shaped nanoparticles with quite a uniform particle size up to 20.6 nm with a few instances of larger particle size and thus confirmed the formation of nanoparticles (Fig. 4). Interestingly, close observation indicated that synthesized nanoparticles were not in direct contact with each other even within aggregates, suggesting stabilization of AgNPs by phytoconstituents.

### EDX analysis

**Fig. 5:**
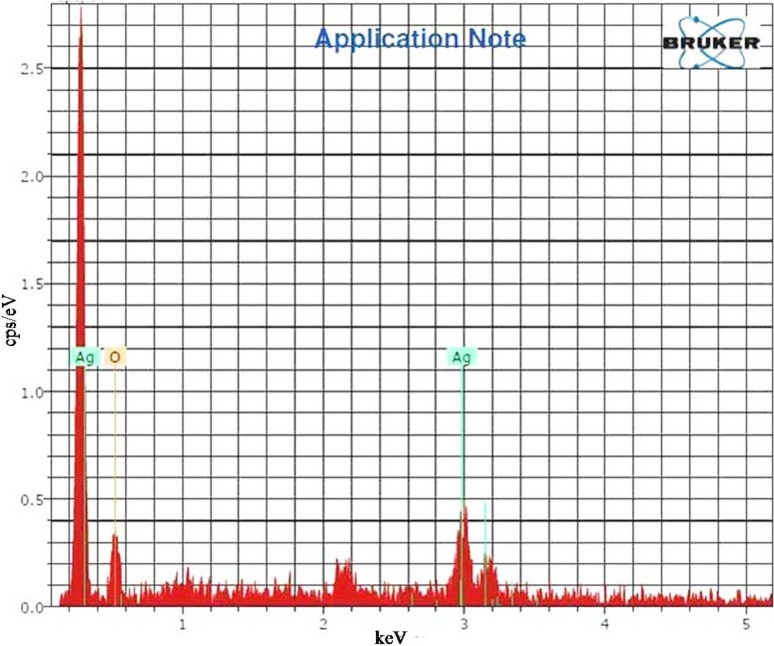
Energy dispersive X-ray spectrum of biosynthesized AgNPs

Presence of elemental silver was revealed by chemical analysis accomplished by means of EDX analysis (Fig. 5). This was due to reduction of silver ions by *TB* extract to AgNPs. The existence of ‘O’ in EDX spectrum might be due to involvement of phytoconstituents in stabilizing AgNPs through ‘O’ related groups (Dauthal and Mukhopadhyay 2013; Ajitha et al. 2015).

### X-ray diffraction

**Fig. 6:**
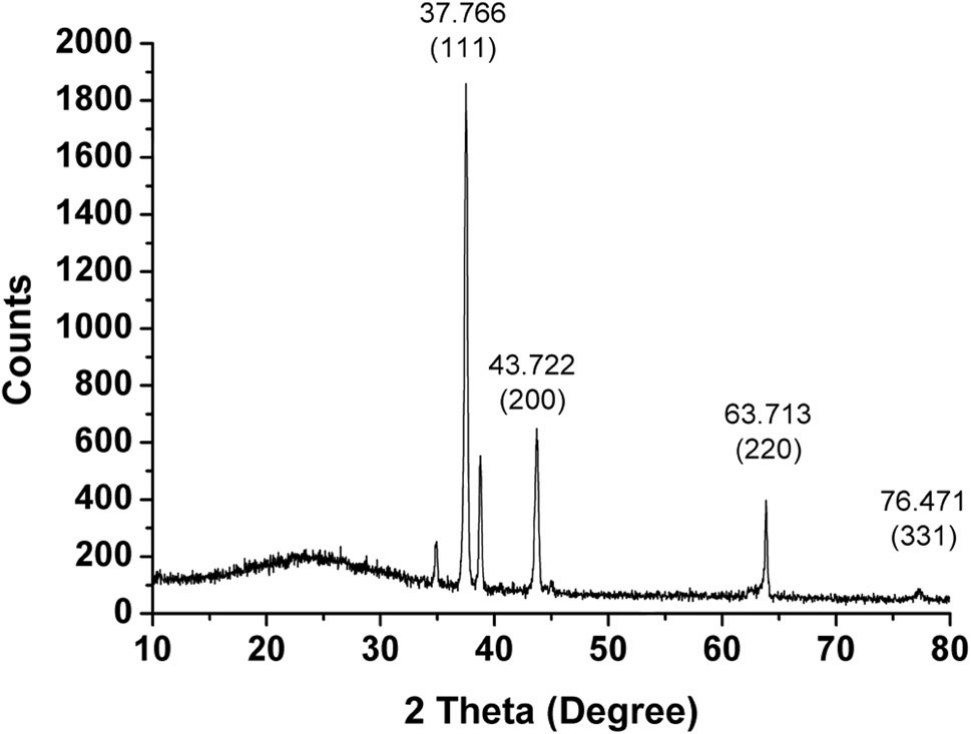
X-ray diffraction pattern of biosynthesized AgNPs

The XRD pattern of the AgNPs (Fig. 6) which showed four intense diffraction peaks at 37.7°, 43.8°, 63.7°, and 76.4° in the whole spectrum of 2*θ* value ranging from 10 to 80 which can be indexed to (111), (200), (220) and (311). These peaks are characteristic of metallic face-centered cubic (FCC) phase of silver and matching with database of Joint Committee on Power Diffraction Standards (JCPDS, 3-065-8428) confirmed the crystalline nature of AgNPs. Some unidentified peaks (34.8°, 38.7° and 45.01°) also appeared in the XRD pattern of AgNPs, which might be due to the phytoconstituents in the extract involved in synthesis and stabilization of the AgNPs.

### Mechanism of formation of AgNPs

**Fig. 7:**
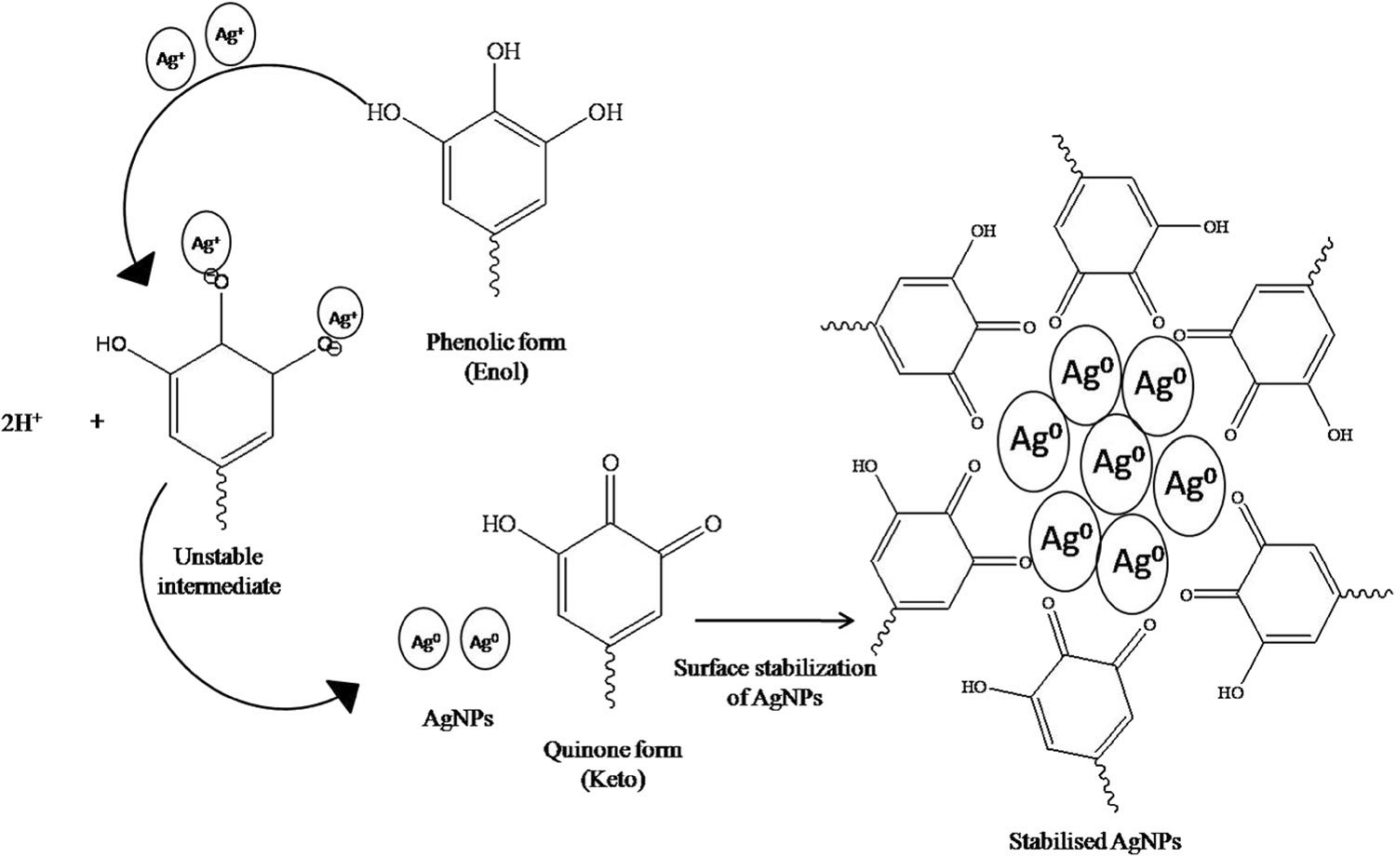
Schematic mechanism of synthesis of AgNPs using polyphenol-rich *TB* extract

The possible mechanism for the synthesis of AgNPs after reduction of Ag^+^ is illustrated in Fig. 7. Polyphenolic compounds can exist in two tautomeric forms; enol (phenolic) and keto (quinine) form. Under alkaline conditions (≥pH 8), the most unstable enolic form of molecule predominates where its –OH group plays a principal role. This form has a strong tendency to donate electrons and undergo oxidation (Basnet and Skalko-Basnet 2011). The Ag^+^ ions form an intermediate complex with –OH, which upon oxidation forms quinone and leads to a subsequent reduction of Ag^+^ ion to Ag^0^ (AgNPs).

### Catalytic activity of AgNPs

**Fig. 8:**
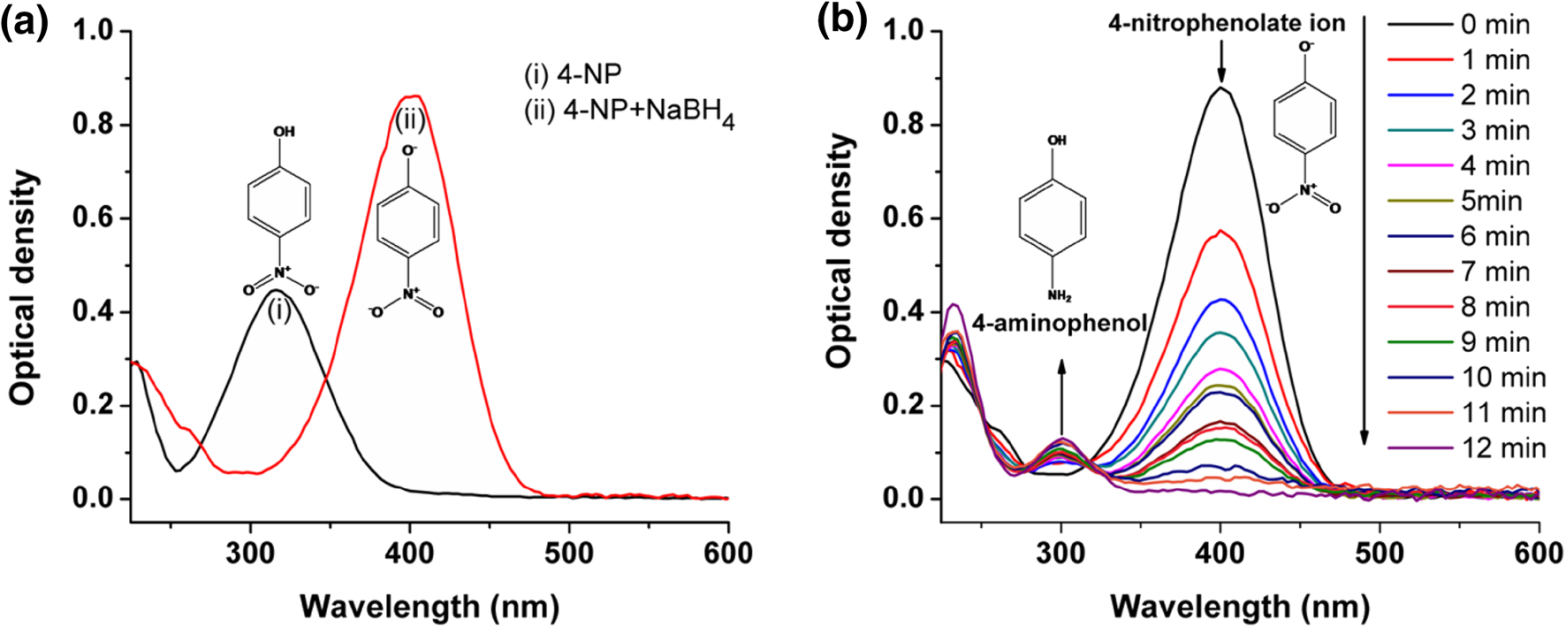
UV–Vis absorption spectra showing **a** formation of 4-nitrophenolate ion from 4-nitrophenol (0.5 mM) in presence of 25 mM NaBH_4_ and **b** catalytic reduction of 4-nitrophenol to 4-aminophenol using AgNPs as a catalyst. Reaction was monitored up to complete reduction of 4-nitrophenol (reaction mixture contained 0.5 mm 4-nitrophenol, 25 mm NaBH_4_ and 0.5% green synthesized AgNPs as catalyst)

In the present study, catalytic action of biosynthesized AgNPs has been evaluated using model reaction, reduction of 4-nitrophenol to 4-aminophenol. The addition of NaBH_4_ to a 4-nitrophenol solution changed the color of solution from light yellow to intense yellow (Online resource Fig. S4) with bathochromic shift from 316 to 400 nm (Fig. 8a). This could be explained by a fact that the addition of NaBH_4_ to a 4-nitrophenol causes a change in pH from acidic to highly basic due to formation of the 4-nitrophenolate ions (Saha et al. 2009). On monitoring this reaction by UV–Vis spectroscopy, it was found that in the presence of only NaBH_4_ intensity of absorption at 400 nm for the 4-nitrophenolate ion remained unchanged even after 30 min (Online Resource Fig. S5). This result confirmed that the reduction of 4-nitrophenol does not proceed without a catalyst. Its reduction was carried out in presence of AgNPs as a catalyst and monitored at different time intervals (Fig. 8b). Very low concentration of AgNPs was used to avoid interference in the absorption of 4-nitrophenolate ion, because both have absorbance at around 400 nm (Online Resource Fig. S6). After the addition of AgNPs to reaction mixture, NaBH_4_ reduced 4-nitrophenol to 4-aminophenol having typical absorption maxima of 298 nm (Chi et al. 2012). The intensity of the absorption peak at 400 nm gradually decreased with time which fully disappeared after ∼12 min while in the meantime, a new absorption peak appeared at 298 nm progressively with increasing intensity (Fig. 8b). However, addition of *TB* extract did not decrease the absorption at 400 nm of 4-nitrophenolate ions which remained unchanged even after 30 min (Online Resource Fig. S7). This result confirmed that *TB* extract did not catalyze the reduction of 4-nitrophenol to 4-aminophenol. The reduction reaction of 4-nitrophenol using AgNPs as catalyst exclusively yielded 4-aminophenol, without any other side products which is evident from existence of isosbestic points (Saha et al. 2009) at 251, 277 and 316 nm in UV–Vis spectra (Fig. 8b).

In this reaction, NaBH_4_ acted as a reducing agent. To achieve complete or maximum reduction of 4-NP, the overall concentration of NaBH_4_ was kept about 50 times higher (25 mM) than 4-nitrophenol (0.50 mM) making this reaction to follow the pseudo-first-order kinetic to determine the catalytic activity of AgNPs.

Catalytic reduction of 4-nitrophenol by NaBH_4_ in presence of AgNPs was a time-dependent process as evident from a plot of concentration of 4-nitrophenol vs. reaction time (Online Resource Fig. S8). Concentration of 4-nitrophenol at any given time in the reaction was calculated from calibration curve of absorbance of 4-nitrophenolate ion at 400 nm vs. respective concentration of 4-nitrophenol.

The rate constant (*k*) of the reduction reaction of 4-nitrophenol using AgNPs as a catalyst was determined from the linear plot of −ln (*A*_t_/*A*_0_) vs. reduction time in seconds (where *A*_*t*_ and *A*_0_ are the concentrations of 4-nitrophenol at time t and 0 s, respectively). It was estimated to be 4.60 × 10^−3^ s^−1^. To compare the catalytic potential of green synthesized AgNPs with previously reported nanomaterials, the activity parameter (*K* = *k*/mass of catalyst) was determined. Volume of AgNPs used in catalytic reduction of 4-nitrophenol was 0.25 mL of 5 mM AgNPs which corresponds to 0.212 mg, thus using this value *k* was calculated and found to be 21.698 s^−1^ g^−1^. Activity parameter of green synthesized AgNPs (in this study) was greater than that of nanomaterials reported earlier (Rashid and Mandal 2007; Chi et al. 2012).

Smaller average particle size (20.74 nm), well monodispersed solution and good stability of the biosynthesized AgNPs attributed to the good catalytic activity of nanoparticles. The smaller size consists of a high surface-to-volume ratio and better exposed Ag atoms on the surface where such atoms act as the potent catalytic sites (Baruah et al. 2013). The reaction mechanism for the reduction of 4-nitrophenol to 4-aminophenol by NaBH_4_ in the presence AgNPs as a catalyst can be well explained by widely accepted Langmuir–Hinshelwood (LH) model (Online Resource Fig. S9). These results indicated that AgNPs might have a significant application in the field of heterogeneous catalysis.

### Antibacterial and antibiofilm potential of AgNPs

**Fig. 9:**
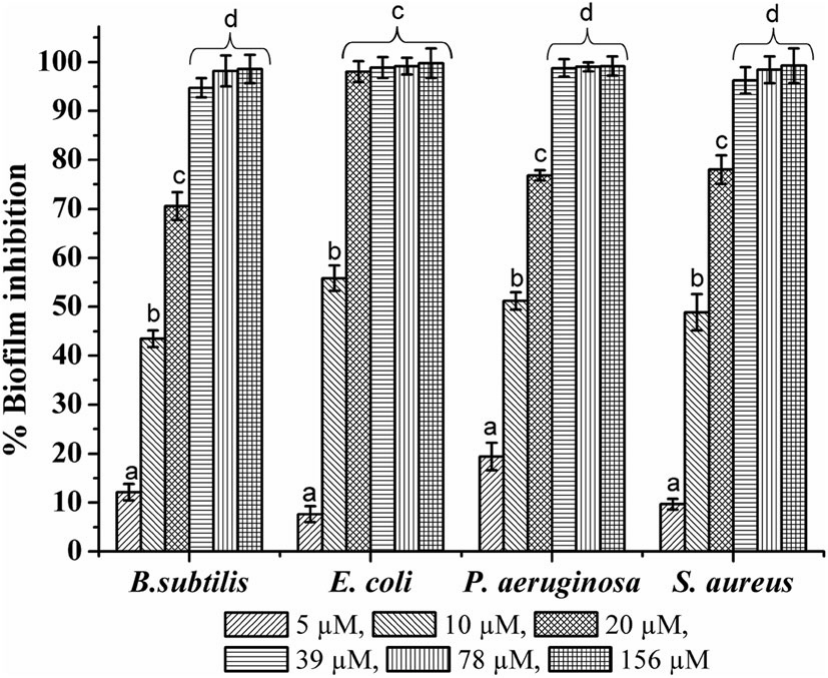
The effect of AgNPs on the formation of bacterial biofilms [values were plotted as a mean ± standard deviation (SD) of *n* = 3 and in a group,* bars* denoted with same letter from ‘*a*–*d*’ do not differ significantly from each other where *P* ≤ 0.05]

The AgNPs synthesized by *TB* extract showed considerable antibacterial activity against the human pathogenic bacterial strains. The zone of inhibition measured (Table 1) suggested that, *P. aeruginosa* was more sensitive to the AgNPs, followed by *E. coli*; while *B. subtilis* and *S. aureus* showed comparatively minimal sensitivity toward the AgNPs. This was also confirmed from the MIC of AgNPs measured against these bacteria (Table 2). *P. aeruginosa* showed the least MIC than others. This could be explained by higher affinity of *P. aeruginosa* cells to colloidal AgNPs than the other tested bacterial strains (Bondarenko et al. 2013). These results showed that AgNPs have a more significant effect on growth of Gram-negative bacteria than that of Gram-positive bacteria. This might be due to differences in the structure and composition of the cell wall of these bacteria (Fayaz et al. 2010). Development of biofilms increases the antibiotic resistance among the microorganisms, which makes it very difficult to control the infections (Mah and O’Toole 2001). The AgNPs have already been effective against planktonic microbial cells; however, the effect of these nanoparticles on formation and eradication of biofilm remains the thrust area. In the present study, the antibiofilm activity of AgNPs was assessed by crystal violet microtiter plate assay. The AgNPs showed higher antibiofilm activity against the Gram-negative bacterial strains than Gram-positive (Fig. 9). In *E. coli* and *P. aeruginosa*, 20 µM and 39 µM concentrations of AgNPs inhibited biofilm formation by more than 98%, respectively. However, in *S. aureus* and *B. subtilis* 78 µM concentrations of AgNPs were needed to inhibit biofilm formation by 98%. This observation may be a result of the structural differences in the composition of the cell wall of these bacteria which supports earlier reports (Martinez-Gutierrez et al. 2013). Among the Gram-negative bacteria, *E. coli* was more susceptible to biofilm inhibitory effect of AgNPs than *P. aeruginosa*. The probable mechanism by which AgNPs reduce/inhibit the formation of biofilms could be an interference or inhibition in the production of extracellular polymeric substances (EPS) by the bacteria (Kalishwaralal et al. 2010) or inhibition of synthesis of quorum-sensing related factors triggering signals in the biofilm formation.

**Table 1:** The antibacterial activity of AgNPs synthesized by using *TB* extract

Sr. no.	Bacteria	Zone of inhibition (mm)*			
		AgNO_3_ 1.25 (mM)	AgNPs 1.25 (mM)	*TB* extract 150 (µg mL^−1^)	Streptomycin 20 µg mL^−1^
1	*B. subtilis*	12.33 ± 0.58	14.33 ± 0.58	ND	32.33 ± 1.53
2	*E. coli*	16.67 ± 1.15	18.67 ± 1.15	ND	37.33 ± 1.15
3	*P. aeruginosa*	18.33 ± 1.15	19.67 ± 0.58	ND	37.33 ± 1.15
4	*S. aureus*	11.67 ± 0.58	14.67 ± 1.53	ND	33.00 ± 2.00

*ND* not detected

* Values were expressed as the mean ± standard deviation (SD) of *n* = 3, with *P* ≤ 0.05 were considered to be statistically significant

**Table 2:** Minimum inhibitory concentrations (MIC) and biofilm inhibitory concentration (BIC) of AgNPs against the tested human pathogenic bacteria

Sr. no.	Bacteria	MIC of AgNPs (µM)*	BIC of AgNPs (µM)*
1	*B. subtilis*	156–313	78
2	*E. coli*	78–156	20
3	*P. aeruginosa*	78–156	39
4	*S. aureus*	156–313	78

* Values were confirmed in triplicate of experiments

**Fig. 10:**
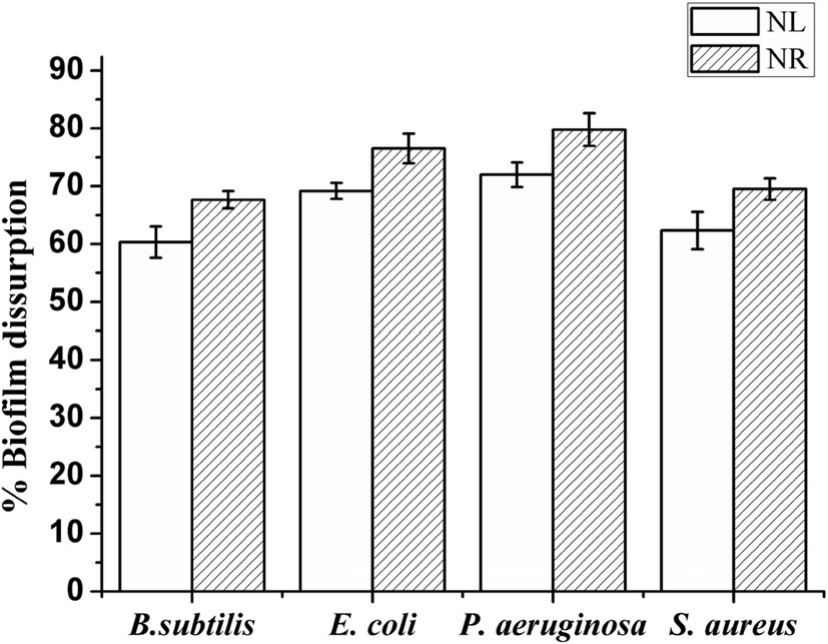
The potential of the AgNPs to disrupt pre-formed biofilms; *NL* nutrient-limited condition and *NR* nutrient-rich condition [values were plotted as the mean ± standard deviation (SD) of *n* = 3]

The ability of the AgNPs to disrupt pre-formed biofilms of selected strains was tested at their BIC. It was found that pre-formed biofilms of all four bacterial strains were significantly disrupted under both nutrient-limited (supplemented with SDW + AgNPs) as well as nutrient-rich (supplemented with media + AgNPs) conditions (Fig. 10). However, greater disruption was observed under nutrient-rich conditions. In biofilm, bacterial growth rate as well as metabolism get reduced or altered. Slow-growing or non-growing cells within the biofilm can shut down their metabolism due to limited nutrient conditions and become resistant to antibacterial treatments (Durmus et al. 2013). Therefore, adding nutrients along with AgNPs to the biofilm can stimulate metabolic microenvironment of the biofilm and may facilitate disruption by better penetration of AgNPs in the biofilm. These results indicated that AgNPs might have a significant application in the field of nanomedicine. However, further studies are needed for their actual implication.

## Conclusion

An efficient and rapid method for the green synthesis of colloidal silver nanoparticles has been established using medicinally important *T. bellirica* fruit aqueous extract. The spectroscopic as well as microscopic properties of biocapped AgNPs were studied. The polyphenolic compounds present in the fruit extract have acted as an effective reducing agent as well as stabilizing agent, resulting in the formation of stable AgNPs of 20.74 nm average size. The synthesized colloidal AgNPs were efficiently used as catalyst for the reduction of 4-nitrophenol to 4-aminophenol which is evident from the spectrophotometric studies. These AgNPs showed potential antimicrobial as well as antibiofilm efficacy against human bacterial pathogens. This work also states the significance of medicinally important plant *T. bellirica* in the development of future AgNP-based nanocatalyst and nanomedicines.

## Electronic supplementary material

Below is the link to the electronic supplementary material.

Supplementary material 1 (DOCX 27,570 kb)
