# Supplementary material for: Instant green synthesis of silver-based herbo-metallic colloidal nanosuspension in Terminalia bellirica fruit aqueous extract for catalytic and antibacterial applications
Source: 3 Biotech. 2017 Apr 13;7(1):36. doi: 10.1007/s13205-016-0589-1 (PMC5391368; doi:10.1007/s13205-016-0589-1)
Supplement: Supplementary file 1 — Supplementary material 1 (DOCX 27,570 kb) [file 13205_2016_589_MOESM1_ESM.docx]

**Electronic supplementary material**

**Title**

**Instant green synthesis of silver based herbo-metallic colloidal nanosuspension in *Terminalia bellirica* fruit aqueous extract for catalytic and antibacterial applications**

**Author’s names**

Sandeep Patil ^1^, Gunjan Chaudhari ^2^, Jayasinh Paradeshi ^1^, Raghunath Mahajan ^3^, Bhushan Chaudhari ^1, *^

^1^ School of Life Sciences, North Maharashtra University, Jalgaon - 425001, India

^2^ Department of Biochemistry, Moolji Jaitha College, Jalgaon - 425002, India

^3^ Post Graduated College of Science Technology and Research, Moolji Jaitha College Campus, Jalgaon- 425002, India

^*^ Corresponding author

**Address for correspondence**-

Bhushan L. Chaudhari, Department of Microbiology, School of Life Sciences, North Maharashtra University, Umavi Nagar, Post Box 80, Jalgaon - 425 001, India.

Tel: +912572257424, Fax: +912572258403, E-mail: [blchaudhari@nmu.ac.in](mailto:blchaudhari@nmu.ac.in), [blchaudhari@hotmail.com](mailto:blchaudhari@hotmail.com)

# Experimental

## Optimization of nanoparticles synthesis

## pH

The pH of this reaction was optimized by using different pH, where the reaction pH was maintained at 2, 4, 6, 7, 8, 10 and 12. The pH was adjusted by using 0.1 M H_2_SO_4_ and 0.1 M NH_4_OH. The absorbance of each resulting solution was measured spectrophotometrically upon 4 fold dilution by deionized distilled water.

### Ratio of concentration of *TB* extract with AgNO_3_ solution

The different ratios of volume of *TB* extract (38.15 ±2.03 mg/mL) with concentration of AgNO_3_ solution were evaluated for synthesis of AgNPs. In this, the ratio of *TB* extract with AgNO_3_ solution was maintained at 1.5:1, 1.5:2, 1.5:3,1.5:4, 1.5:5 and 1.5:6 (volume in mL: mM) for a reaction volume of 100 ml. The absorbance of the resulting solutions was measured spectrophotometrically with 4 fold dilution by deionized distilled water.

### Time

The time of microwave irradiation was optimized by using different time intervals, where the reaction time was monitored from 0 minute to 5 minute. The absorbance of the resulting solutions was measured spectrophotometrically with 4 fold dilution by deionized distilled water.

**
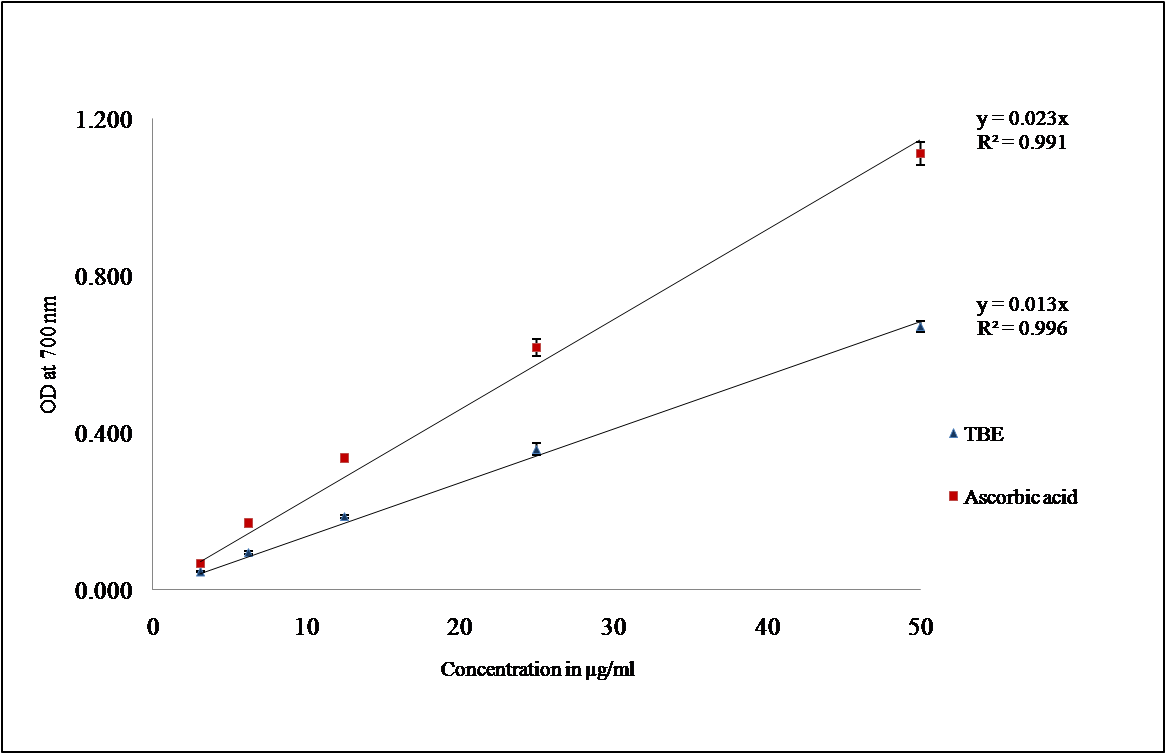
**

**Figure S1:** The Ferric ion reducing power of TBE compared with ascorbic acid. Increase in absorbance of sample indicates increase in reducing power. Data is presented as means ± SD (n=6).

**
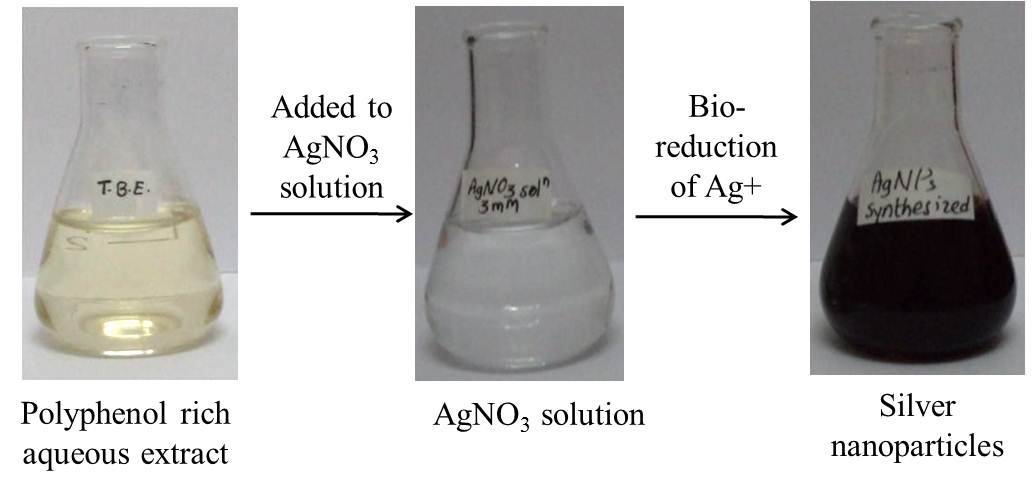
 Figure S2**: Polyphenol rich *Terminalia bellirica* fruit aqueous extract mediated synthesis of AgNPs as evident from development of dark brown color.

**
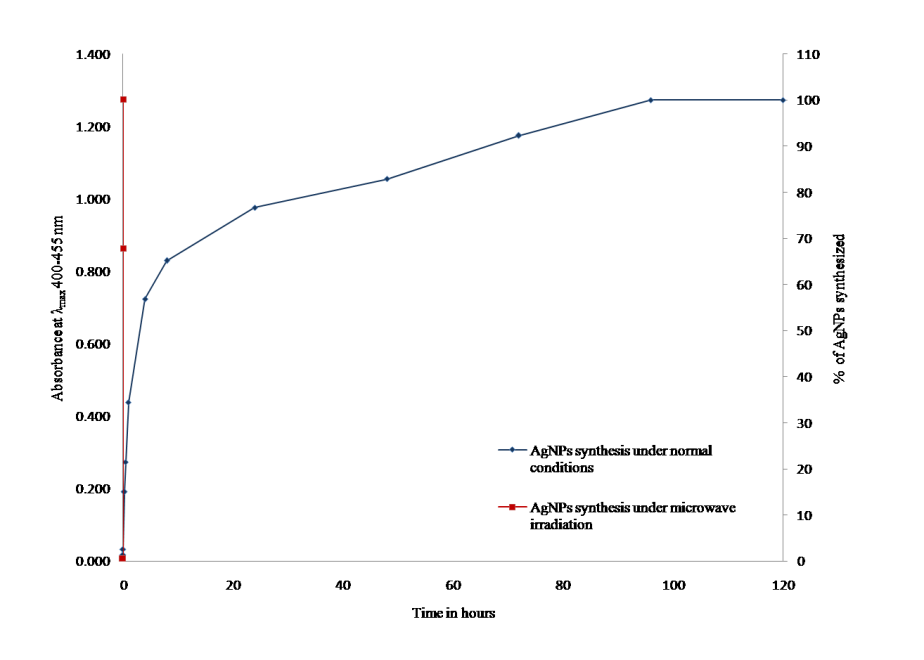
**

**Figure S3:** A plot of absorption of the reaction mixture at 400-450 nm as a function of time (after AgNPs synthesis under normal conditions and microwave irradiation); Data is presented as means ± SD (n=3).

**
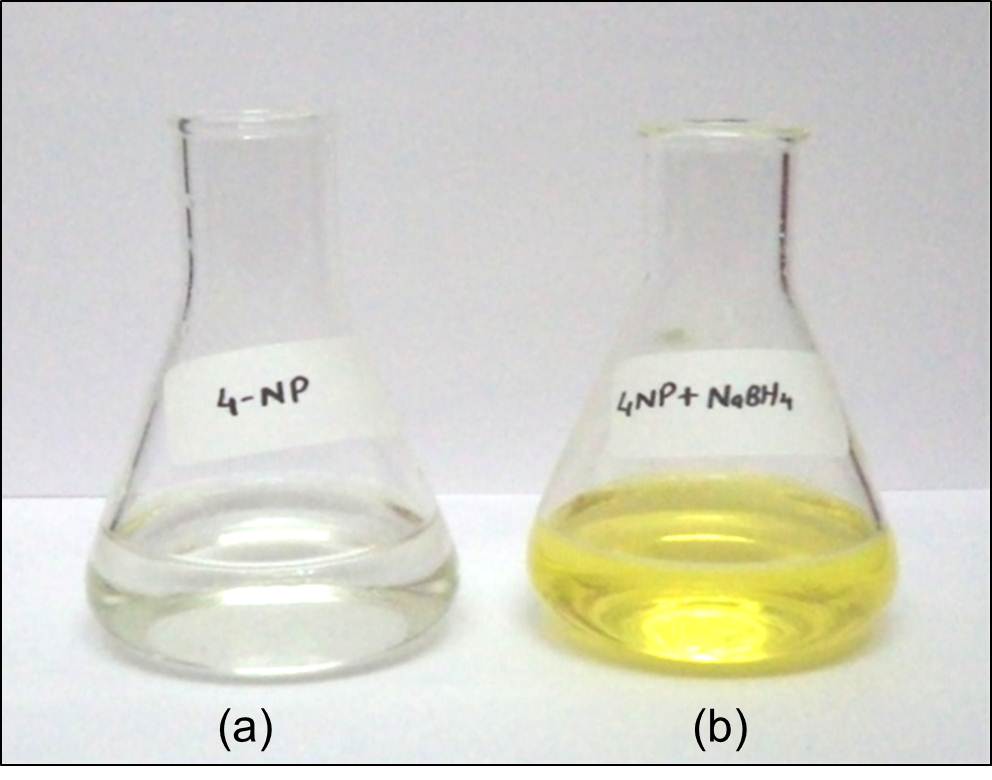
**

**Figure S4:** Development of intense yellow color from light yellow after the addition of NaBH_4_ to a 4-nitrophenol solution forming 4-nitrophenolate ion

**

**

**Figure S5:** UV-vis absorption spectra of reaction mixture of 0.5 mM 4-nitrophenol and 25 mM NaBH_4_ with no catalyst added, reaction monitored for 30 minutes.

**
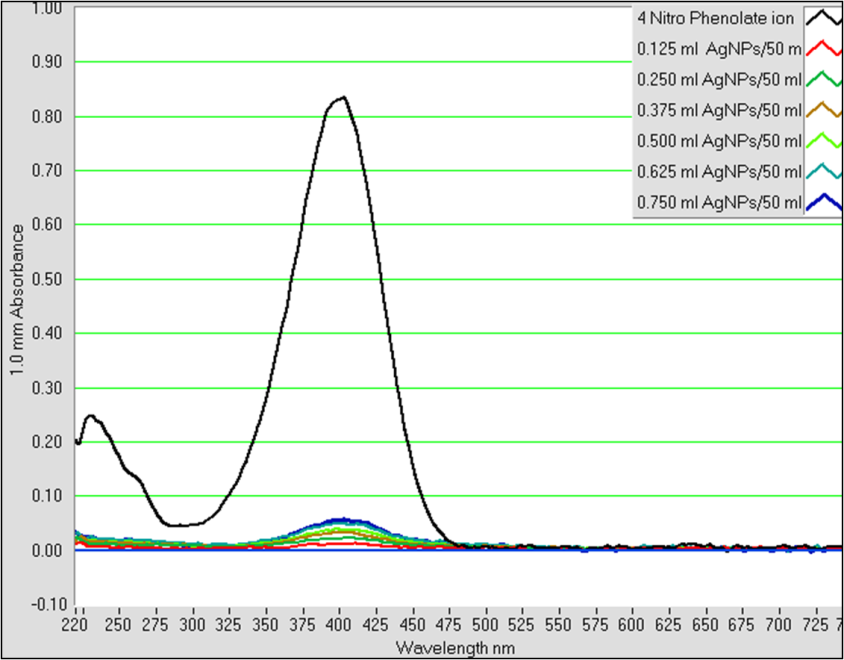
**

**Figure S6:** Addition of very low conc. of AgNPs does not have interference in absorption of 4- nitrophenolate ion at around 400 nm.

**

**

**Figure S7:** Addition of *TB* extract did not decrease the absorption of 4-nitrophenolate ions at 400 nm which remained unchanged even after 30 min

**
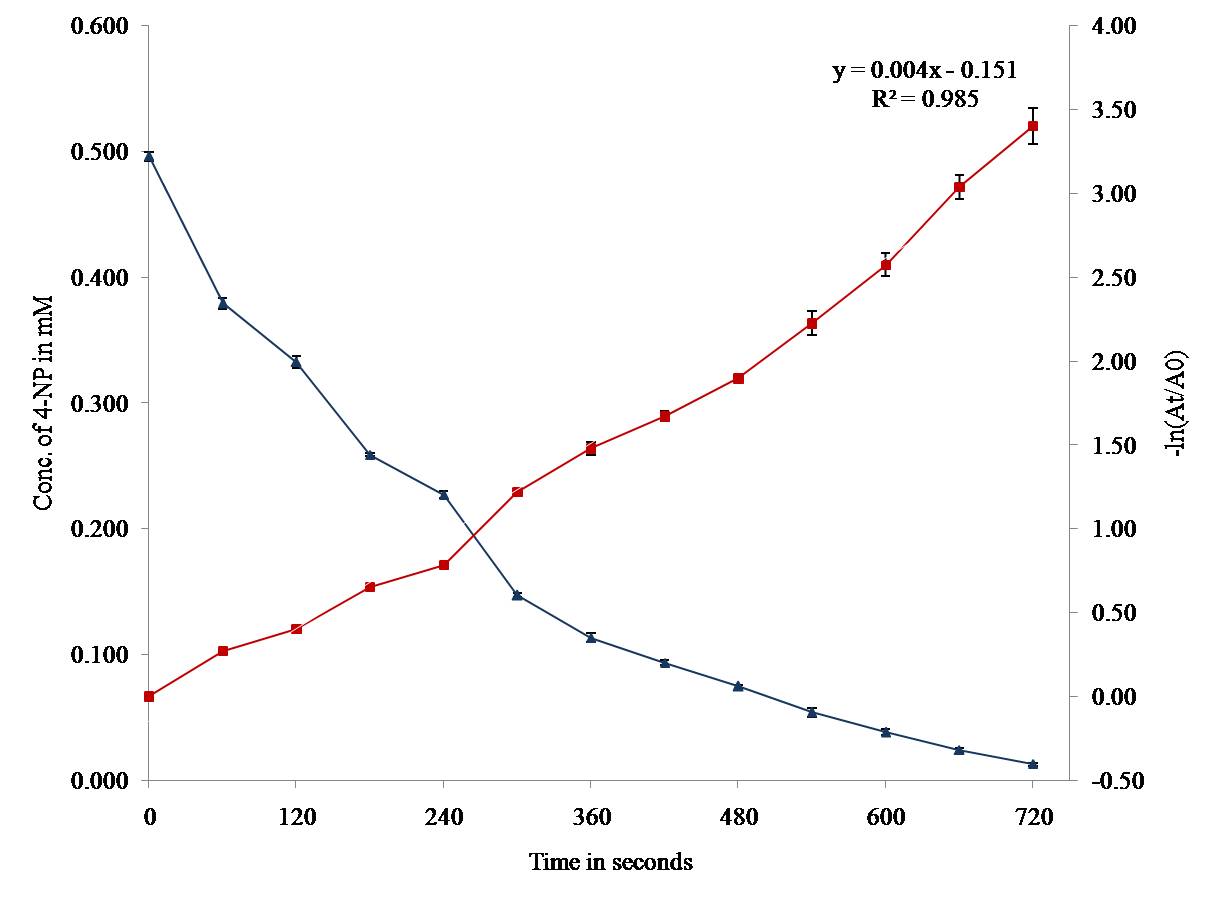
**

**Figure S8:** A plot of (a) concentration of 4-nitrophenol vs. time and (b) –ln (A_t_/A_0_) vs. time; for the reduction reaction of 4-nitrophenol by excess NaBH_4_ in presence of AgNPs as a catalyst following pseudo first order reaction kinetics.


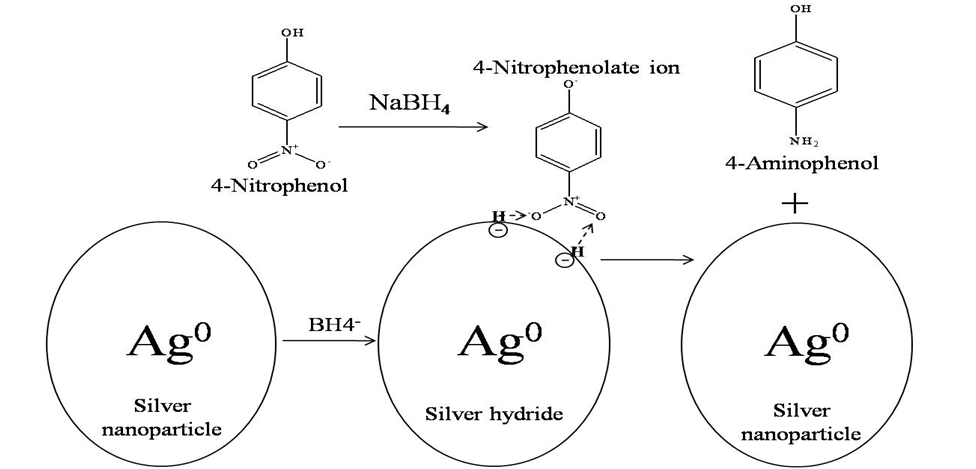


**Figure S9:** Schematic representation of model for the mechanism of the reduction of 4-nitrophenol by NaBH_4_ in the presence of AgNPs by Langmuir-Hinshelwood model. Langmuir−Hinshelwood (LH) model is widely accepted to explain the reaction mechanism for the reduction of 4-nitrophenol to 4-aminophenol by NaBH_4_ in the presence nanomaterials as a catalyst schematically shown in Figure S4. Adsorption of borohydride ions occur on the surface of AgNPs and transfer a hydrogen species to the surface of the nanoparticles. Simultaneously, 4-nitrophenol molecules are adsorbed on the surface of the nanoparticles. Adsorption of both borohydride ions and 4-nitrophenol molecules is a reversible process. Moreover, the adsorption or desorption equilibrium and diffusion of reactants to the nanoparticles are considered to be fast. The 4-nitrophenol adsorbed to AgNPs react with the hydrogen atom bound to the surface of AgNPs leading to the reduction of 4-nitrophenol to 4-aminophenol. Synthesized 4-aminophenol is released from the surface of AgNPs while AgNPs surface is free and available for next cycle of catalysis. This mechanism could be applied to resolve the reduction of 4-nitrophenol to 4- aminophenol in presence of biosynthesized AgNPs.
